# Imaginary Companions, Inner Speech, and Auditory Verbal Hallucinations: What Are the Relations?

**DOI:** 10.3389/fpsyg.2019.01665

**Published:** 2019-07-30

**Authors:** Charles Fernyhough, Ashley Watson, Marco Bernini, Peter Moseley, Ben Alderson-Day

**Affiliations:** ^1^Department of Psychology, Durham University, Durham, United Kingdom; ^2^Department of English Studies, Durham University, Durham, United Kingdom; ^3^School of Psychology, University of Central Lancashire, Preston, United Kingdom

**Keywords:** hallucination proneness, signal detection, theory of mind, social cognition, imagination, development

## Abstract

Interacting with imaginary companions (ICs) is now considered a natural part of childhood for many children, and has been associated with a range of positive developmental outcomes. Recent research has explored how the phenomenon of ICs in childhood and adulthood relates to the more unusual experience of hearing voices (or auditory verbal hallucinations, AVH). Specifically, parallels have been drawn between the varied phenomenology of the two kinds of experience, including the issues of quasi-perceptual vividness and autonomy/control. One line of research has explored how ICs might arise through the internalization of linguistically mediated social exchanges to form dialogic inner speech. We present data from two studies on the relation between ICs in childhood and adulthood and the experience of inner speech. In the first, a large community sample of adults (*N* = 1,472) completed online the new Varieties of Inner Speech – Revised (VISQ-R) questionnaire (Alderson-Day et al., 2018) on the phenomenology of inner speech, in addition to providing data on ICs and AVH. The results showed differences in inner speech phenomenology in individuals with a history of ICs, with higher scores on the Dialogic, Evaluative, and Other Voices subscales of the VISQ-R. In the second study, a smaller community sample of adults (*N* = 48) completed an auditory signal detection task as well as providing data on ICs and AVH. In addition to scoring higher on AVH proneness, individuals with a history of ICs showed reduced sensitivity to detecting speech in white noise as well as a bias toward detecting it. The latter finding mirrored a pattern previously found in both clinical and nonclinical individuals with AVH. These findings are consistent with the view that ICs represent a hallucination-like experience in childhood and adulthood which shows meaningful developmental relations with the experience of inner speech.

## Introduction

Between a third and two-thirds of young school-age children will engage with imaginary companions (ICs), defined as invisible characters with whom children converse and interact (Taylor et al., 2004). These characters can include invisible characters which nevertheless have an air of reality for the child (Svendsen, 1934), and personified objects (imaginary beings that are embodied in a toy or object). Since research in this area adopted new methodological standards in the 1990s, ICs have been associated with a range of positive developmental outcomes (Taylor, 1999). Several studies have linked engagement with an IC to superior social cognition (Taylor and Carlson, 1997; Roby and Kidd, 2008; Davis et al., 2011), while other studies have indicated that children with an IC are more creative (Schaefer, 1969; Seiffge-Krenke, 1997; Hoff, 2005), more sociable (Mauro, 1991), and capable of constructing more complex narratives (Trionfi and Reese, 2009).

Historically, however, engaging with ICs has been considered a cause for concern, and even a possible marker of future mental illness. Although this view has now been discredited (Taylor, 1999), several features of engaging with ICs raise parallels with an experience that is often considered pathological: the experience of auditory verbal hallucinations (AVH) or “hearing voices.” Hallucinations are defined as percept-like experiences which occur in the absence of an appropriate stimulus, which have the full force or impact of the corresponding veridical perception, and which are not under the experiencer’s direct or voluntary control (Slade and Bentall, 1988). Several researchers have considered whether the experience of engaging with ICs bears commonalities with that of AVH. Intuitively, a point of commonality should reside in the fact that both ICs and AVH generate disembodied yet percept-like social agents with whom to interact. Unlike ICs, however, AVH are not usually experienced as willfully created by the subject, but rather as spontaneously occurring emergences of quasi-perceptual agents (Nayani and David, 1996; Woods et al., 2015). ICs might also be assumed to show cooperative and positive interactional social behavior compared to AVH, which often have a negative emotional valence. In addition, AVH can be perceived as located either internally or externally in space, whereas ICs usually tend to be projected as agents in the external world. In short, there seem to be good reasons for testing productive comparisons between AVH and ICs, and research and theoretical insights on the latter might inform and challenge theoretical and empirical work on the former.

One line of research has examined engagement with ICs as involving non-veridical percept-like experiences. Pearson et al. (2001) found that children’s reporting of ICs in middle childhood related to their tendency to report hearing words in an ambiguous auditory stimulus. Using a more rigorous methodology, Fernyhough et al. (2007) replicated this effect in two samples, linking the childhood experience of ICs to a Vygotskian view of development by which thinking develops through the gradual internalization of linguistically mediated social exchanges to form inner speech (see Alderson-Day and Fernyhough, 2015, for a review). This interpretation was subsequently supported by Davis et al.’s (2013) finding that children with ICs were more advanced (relative to their peers without ICs) on the internalization of private speech, considered by Vygotsky to be a precursor of inner speech.

Another line of research has considered the extent to which ICs are under the experiencer’s voluntary control. There is growing recognition that the behavior of ICs is not always under children’s control, providing a further rationale for considering at least some manifestations of ICs as hallucination-like phenomena. Hoff (2005) and Taylor et al. (2007) have presented findings suggesting considerable variability in the extent to which children report that their ICs can have alternative thoughts, feelings, or/and behaviors to their own. Taylor and colleagues have referred to this as the “illusion of independent agency.” In this article, we use the equivalent term IC *autonomy* to refer to IC behaviors that are not compliant with the host’s own cognitions, emotions, and intentions.

A further way in which research into ICs has developed in recent years concerns a growing recognition that engagement with ICs can persist into adulthood. Taylor et al. (2004) found continued engagement with ICs (in a sample that had originally been studied in the preschool years) at age 7 and on into adolescence. Although anecdotal evidence suggests that some adults engage with ICs (Taylor, 1999), to date, there has been no systematic study of the persistence of ICs into adulthood. Beyond the question of the continued engagement with ICs in adulthood, another avenue of research involves examining what – if any – cognitive differences in adulthood may be observed in those with a history of ICs. For example, Firth et al. (2015) found that adults reporting having had an IC in childhood scored more highly on a scene construction task, employed as an objective measure of imaginative capacity, as well as rating themselves as more imaginative.

We set out to explore several hypotheses concerning the relations between ICs and hallucinatory experiences. In the first study, we asked a large sample of online respondents about their experience of ICs in childhood and adulthood. In line with the reasoning of Pearson et al. (2001) and Fernyhough et al. (2007), we predicted that individuals reporting engagement with ICs would show greater susceptibility to hallucination-like experiences in adulthood. We additionally took measures of the sensory vividness of reported IC interactions and IC autonomy.

We also examined ideas from Fernyhough et al. (2007) and Davis et al. (2013) on the relation between ICs and the development of inner speech. Using a new questionnaire assessment of the quality of inner speech in adulthood, we investigated relations among IC status, varieties of inner speech, and hallucination proneness in our large sample of online respondents. Specifically, we predicted that those with experience of ICs would evidence more expanded, social-like experiences of inner speech, such as reporting other people in inner speech, or inner speech with dialogic characteristics. We also gathered, in the largest sample examined to date, novel data on the persistence of ICs into adulthood.

In the second study, we worked with a smaller, separate sample of participants to explore the cognitive processes involved in distinguishing real events from imagined ones. We assessed this capacity with an auditory signal detection paradigm. Biased performance on such tasks has been linked to reality-monitoring processes and strongly implicated in the experience of AVH (Bentall, 1990; Brookwell et al., 2013), but has never previously been examined in relation to IC engagement. We also assessed social cognition (theory of mind) to test specificity of any cognitive effects.

## Study 1: Method

### Participants

A sample of 1,472 participants (age *M* = 38.84; SD = 13.42; 1,112 females) were recruited *via* an online survey originally designed to explore inner speech and reading imagery (Alderson-Day et al., 2017). The survey was advertised *via* a UK national newspaper (*The Guardian*) and the Edinburgh International Book Festival. The majority of participants were based in the UK (*n* = 748) or USA (*n* = 213) and education levels were high, with over 80% of the sample possessing a graduate degree or above (for a full description of the sample, see Alderson-Day et al., 2018).

### Measures

#### Imaginary Companions Questionnaire

Due to the lack of measures for assessing ICs in adulthood, a bespoke schedule of questions was devised to assess IC status (past and current), plus characteristics of IC experiences (see Table 1).

**Table 1:** Imaginary companion incidence and characteristics.

*n* (%)					
1. Did you ever have any imaginary friends when you were growing up?			Yes	No	NA/missing
			608 (41%)	859 (58%)	5 (<1%)
2. Do you have any imaginary friends now?			Yes	No	NA/missing
			110 (7%)	1,358 (92%)	4 (<1%)
3. If you have had an imaginary friend, did you ever hear their voice?					
Never	Very occasionally	Some of the time	Most of the time	All of the time	NA/missing
198 (31%)	97(15%)	140 (22%)	94 (15%)	57 (9%)	46 (7%)
4. If you have had an imaginary friend or friends, did you ever see them or have other sensory experiences in relation to them?					
Never	Very occasionally	Some of the time	Most of the time	All of the time	NA/missing
249 (39%)	131 (21%)	120 (19%)	67 (11%)	29 (5%)	36 (6%)
5. If you ever had an imaginary friend or friends, did they sometimes act of their own accord (as opposed to always doing what you told them to do)?			Yes	No	NA/missing
			242 (38%)	307 (49%)	83 (13%)

*Percentages for items 3–5 were calculated from the total of all participants who had an imaginary companion at some point (n = 632)*.

The following questions were used:

1. Did you ever have any imaginary friends when you were growing up?
2. Do you have any imaginary friends now?
3. If you have had an imaginary friend, did you ever hear their voice?
4. If you have had an imaginary friend or friends, did you ever see them or have other sensory experiences in relation to them?
5. If you ever had an imaginary friend or friends, did they sometimes act of their own accord (as opposed to always doing what you told them to do)?

Questions 1, 2 and 5 were answered with a yes/no response. Questions 3 and 4 were completed with the following response options: *Never, Very occasionally, Some of the time, Most of the time, All of the time*.

#### Launay-Slade Hallucination Scale – Revised, Auditory Subscale

A 5-item version of the Launay-Slade Hallucination Scale (henceforth LSHS-A) was chosen to examine proneness to auditory hallucination-like experiences (“hearing voices”) in the sample (Bentall and Slade, 1985; Morrison et al., 2000). Participants answer items describing a range of perceptual errors (such as hearing one’s name being called momentarily) and rate their frequency from 1 (*Never*) to 4 (*Almost Always*). Despite being a short measure, the 5-item LSHS-A has moderate/good internal reliability (Cronbach’s alpha = 0.69; McCarthy-Jones and Fernyhough, 2011). Online assessment of psychopathological variables has been shown to be reliable compared to traditional pen-and-paper methods (Jones et al., 2008).

#### Varieties of Inner Speech Questionnaire – Revised

The Varieties of Inner Speech Questionnaire – Revised (VISQ-R) is a 26-item scale that requires participants to report on the frequency of various phenomenological characteristics of inner speech (Alderson-Day et al., 2018). It has five factors: dialogic inner speech, evaluative/critical inner speech, condensed inner speech, other people in inner speech, and positive/regulatory inner speech. The scale has strong internal reliability (alphas > 0.8) and is consistently related to various psychopathological traits, such as hallucination proneness, dissociation, anxiety, and depression (Alderson-Day et al., 2018).

### Analysis

All data were analyzed in R, unless otherwise stated. Differences in hallucination proneness and inner speech characteristics were compared between four groups based on their IC status: those with no history of having an IC, those with a childhood IC only, those with a childhood and current IC, and those with a current IC only. For inferential statistics, skewed distributions were corrected using either log transformations (LSHS, Condensed VISQ, Other People VISQ) or square root transformations of reflected scores (Dialogic VISQ, Evaluative VISQ). For ease of interpretation, all figures and tables report untransformed scores. No transformed outcomes failed Levene’s test (all *p* > 0.05).

## Study 1: Results

### Characteristics of Imaginary Companions

Table 1 displays the main IC characteristics reported across the sample. The majority – 56% – of participants had never had any experience of an IC, but as many as 41% had an IC in childhood. A total of 69% of participants with an IC (at any point in their life) reported having had an experience of hearing the IC’s voice on at least one occasion, while 61% had had visual or other sensory experiences. From those who responded to both questions 1 and 2 of the survey (*N* = 1,463), the four groups separated out as follows: those with no history of an IC (*n* = 831), those with a childhood IC only (*n* = 522), those with a childhood and current IC (*n* = 84), and those with a current IC only (*n* = 26). Table 2 displays mean ages and scores for hallucination proneness (LSHS-R) and inner speech characteristics (the VISQ-R) across the four groups.

**Table 2:** Hallucination proneness and inner speech features by imaginary companion status.

		*M* (SD)							
		No IC		Childhood IC only		Childhood and current IC		Current IC only	
Age		38.77	(13.07)	39.08	(13.83)	37.49	(13.84)	40.15	(14.2)
LSHS-A		8.76	(2.77)	9.29	(2.87)	10.56	(3.6)	8.58	(2.79)
VISQ-R	Dialogic	23.69	(6.88)	24.94	(6.69)	27.11	(5.84)	23.24	(6.73)
	Evaluative	33.15	(8.34)	34.02	(8.51)	36.91	(7.06)	33.42	(10.40)
	Other people	13.93	(7.34)	15.92	(7.67)	19.30	(8.05)	15.91	(8.10)
	Condensed	14.33	(6.61)	14.24	(6.21)	13.98	(6.64)	17.62	(7.00)
	Positive	18.07	(4.88)	18.61	(4.65)	19.62	(5.34)	18.76	(5.00)

*IC, Imaginary companion; LSHS-A, Launay-Slade Hallucination Scale – Auditory; VISQ-R, Varieties of Inner Speech Questionnaire – Revised*.

### Relations With Hallucination Proneness and Inner Speech

**Figure 1:**
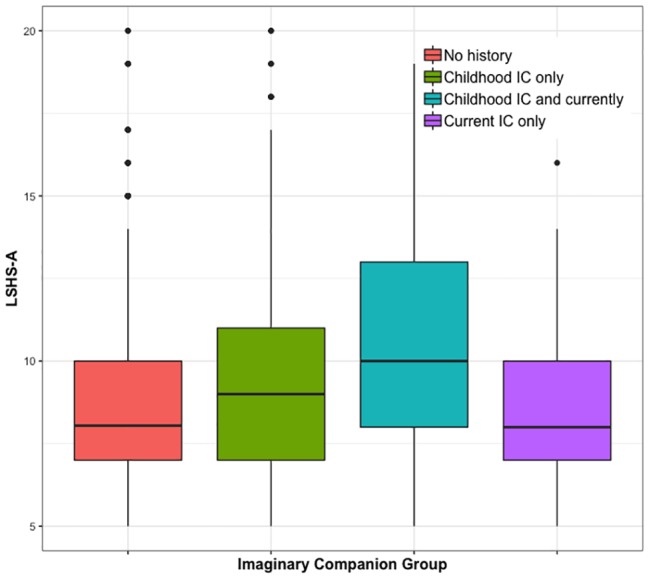
Hallucination proneness by imaginary companion group.

As can be seen in Figure 1, scores for LSHS-A were positively skewed, with a majority of participants across all groups reporting very little experience of hallucinations. Nevertheless, a one-way ANOVA on log-transformed scores for the LSHS-A indicated a significant main effect of group, *F*(3, 1,459) = 10.74, *p* < 0.001, $\eta_{p}^{2}$ = 0.022. *Post hoc* Games-Howell tests (which correct for multiple comparisons) indicated that those with a childhood and current IC scored higher for hallucination proneness than all three other groups (all *p* < 0.044), while those with a past IC were also more hallucination prone than participants with no IC at all (*p* = 0.002).

With an alpha correction to 0.01 (to account for multiple testing across the five VISQ-R subscales), similar results were observed for dialogic inner speech, *F*(3, 1,459) = 9.15, *p* < 0.001, $\eta_{p}^{2}$ = 0.018; evaluative/critical inner speech, *F*(3, 1,459) = 5.58, *p* = 0.001, $\eta_{p}^{2}$ = 0.011; and other people in inner speech *F*(3, 1,459) = 15.84, *p* < 0.001, $\eta_{p}^{2}$ = 0.032. Differences in positive inner speech were marginal but non-significant, *F*(3, 1,459) = 3.43, *p* = 0.016, $\eta_{p}^{2}$ = 0.007, while no group differences were observed for condensed inner speech, *F*(3, 1,459) = 1.96, *p* = 0.117, $\eta_{p}^{2}$ = 0.004.

Broadly similar pairwise differences were observed in *post hoc* analysis, again using Games-Howell tests. For dialogic inner speech, those with a past and current IC scored higher than those without an IC (*p* < 0.001) and those with only a childhood IC (*p* = 0.019), but not those with a current IC only (*p* = 0.051), while more dialogic inner speech was also observed in those with a childhood IC compared to those with no IC history (*p* = 0.005). The same pattern of group comparisons was evident for the Other People inner speech factor (all *p* < 0.01). For evaluative/critical inner speech, scores only significantly differed between those with both current and childhood ICs compared to the childhood IC group (*p* = 0.019) and those with no IC (*p* < 0.001).

## Study 1: Discussion

Study 1 set out to explore for the first time relations between IC status in childhood and adulthood, the quality of inner speech, and proneness to AVH. Data gathered from a large sample of online respondents supported predictions that experience of ICs would be associated with a greater susceptibility to AVH. The highest scores for AVH proneness were observed in those who had both had an IC in childhood and continued to have one in adulthood. Comparable findings were observed in relation to measures of social-like experiences of inner speech, particularly on the Dialogic, Other People, and Evaluative/Critical factors.

A further aim of Study 1 was to gather novel data on the persistence of ICs into adulthood, using the largest sample employed to date in such analyses. The proportion of individuals reporting experience of ICs in childhood (41%) was roughly in line with previous studies. A total of 110 participants (representing around 7.5% of the sample) reported experience of ICs in adulthood. Of those reporting a childhood IC, 13.8% reported continued IC engagement in adulthood. Our findings are also consistent with previous observations that the behavior of ICs is not always fully under the experiencer’s control (Taylor et al., 2007).

Several limitations of Study 1 need to be mentioned. Although the group differences reached high levels of significance, they represent what would conventionally be described as small effects ($\eta_{p}^{2}$ between 0.011 and 0.032). Further limitations were the embedding of our data collection within a wider study of reading imagery (Alderson-Day et al., 2017), and the exclusive reliance on online self-report as a method of data gathering. The consequence of the former is that this sample may be skewed toward those high in imagery vividness and imaginative tendencies in the general population, while the latter limitation might have served to increase correlations among variables within the sample (i.e., common-method variance). Accordingly, in our second study, we used cognitive tasks that have previously been associated with the presence of hallucinations to obtain arguably more objective measures of relevant processes. The fact that we were working with a smaller sample also allowed us to obtain parental corroboration of childhood IC status, which is considered best practice in IC research (Taylor and Carlson, 1997).

## Study 2: Method

Employing a smaller sample of participants in a lab-based study, we replicated the measures used in Study 1 and added two tasks to assess cognitive processes previously implicated in IC status and AVH proneness. One such task is auditory signal detection, a measure which requires participants to detect speech clips embedded in noise. Previous findings have indicated that AVH proneness is associated with a tendency to falsely detect speech in noise, with signal detection parameters indicating that this is due to a response bias, rather than reduced task sensitivity. This has been linked to reality monitoring processes (i.e., the processes used to distinguish between self- and non-self-generated stimuli; Brookwell et al., 2013), or the influence of top-down processes on perception (Moseley et al., 2016), and has never previously been examined in relation to IC engagement. The second process, social cognition or theory of mind, has been associated with IC status in childhood, but has not been consistently associated with hallucination proneness in adult population samples (e.g., Fernyhough et al., 2008). To further examine the role of theory of mind, we therefore included a commonly used measure of “mentalizing” abilities: the Reading the Mind in the Eyes test (Baron-Cohen et al., 2001).

### Participants

A sample of 14 adults with a history of imaginary companions (age *M* = 21.21, SD = 2.26, 4 males) and 34 adults with no imaginary companions (age *M* = 21.18, SD = 2.18, 13 males) were recruited *via* a university participant pool, email circular, social media, and a recruitment blog article (Watson, 2017). Participants received either course credit or a gift voucher for their participation. On recruitment into the study, participants were asked to complete a short schedule about their history of imaginary companions (see Table 3), and to ask their parents to complete three questions: whether their child (1) had an IC when they were younger, (2) spoke to the IC, or (3) actively played with the IC. No participants who reported an IC failed the parental verification check; however, two participants did not recall having an IC when their parents reported that they had (including outwardly interacting with the IC). The latter two participants were included in the IC group, but were marked in later analysis in case they unduly influenced the group results.

**Table 3:** Participant IC schedule and response frequencies in IC group (*n* = 14).

Participant questions	Yes (%)
1. Did you have an imaginary friend during childhood?	85.7^*^
2. Did you speak to this imaginary friend?	85.7
3. Did you actively play with this imaginary friend?	78.6
4. Do you have any imaginary friends now?	0
5. If you ever had an imaginary friend, did they sometimes act of their own accord (as opposed to always doing what you told them to do)?	21.4

* *Two participants were included because their parents reported them having an IC in childhood, even though they did not recall having one*.

### Measures

#### Imaginary Companions Questionnaire

Table 3 shows the questions asked of participants about their IC history. The questions used were broadly similar to those used in Study 1, although specific questions about observable behaviors (e.g., playing with the IC) were also included to allow for comparison with parent reports. Each question was answered with a binary response (*Yes* or *No*).

#### Launay-Slade Hallucination Scale – Revised

For Study 2, a 9-item version of the LSHS was used which incorporated the five auditory LSHS items used in Study 1, and added four items from the full LSHS relating to visual experiences (for example, *I see shadows and shapes when there is nothing there*) (Bentall and Slade, 1985; Morrison et al., 2000). The longer scale provides a more reliable estimate of hallucination proneness, and is in line with use of the LSHS in the wider hallucinations literature (which often focuses on general hallucination proneness; e.g., Siddi et al., 2019).

#### Signal Detection Task

An auditory signal detection task (SDT) was used modeled on those used by Smailes et al. (2014) and Moseley et al. (2014). Participants were asked to listen to 60 trials containing 5-s bursts of white noise, played over headphones. In 12 trials, speech was clearly present in the white noise at an audible volume; in 24 trials, no speech was present; and in 24 trials, speech was played at a threshold volume calibrated in piloting to allow a 50% success rate (pilot sample *n* = 10). The speech was identical to that employed in previous studies and first used by Barkus et al. (2007): a 1.5-s clip of a male voice reading aloud from an instruction manual. On each trial, participants were asked to indicate whether speech was present or absent, providing four response outcomes: *hits* (correctly identifying speech when present), *misses* (failing to identify speech when present), *correct rejections* (identifying when speech is absent), and *false alarms* (hearing speech when none is being played). Following Stanislaw and Todorov (1999), these outcomes were used to calculate beta (*β*), a measure of response bias, and d-prime (*d*′), a measure of sensitivity or discrimination. Scores below 1 for beta indicate a bias toward classifying trials as containing speech, while scores above 1 indicate a bias away from identifying speech. Higher scores on discrimination indicate better sensitivity on the task. Following previous studies of hallucination proneness and signal detection, the primary outcome on the task was beta (see Brookwell et al., 2013, for a review), while *d*′ – on which people with hallucinations usually do not differ from control participants – was used as a control outcome.

#### Reading the Mind in the Eyes Task

This social cognition (theory of mind) task was used as a control task to determine specificity of any effects relating to signal detection (Baron-Cohen et al., 2001). The revised adult version (Baron-Cohen et al., 2001) was used to accommodate the age of the sample and was presented in printed form. Both validity and test-retest reliability have been found to be high enough to treat scores as a good approximation of theory of mind ability (including cross-culturally). Participants were asked to select one of four words that they believed best described the emotional or mental state of 30 different sets of eyes. The selection of words varied for each question. Definitions were available for each participant, including an example sentence. All participants reported being proficient in English.

### Procedure

All testing took place in a quiet room away from auditory distractors. Following consent, participants completed the LSHS and a paper version of the Reading the Mind in the Eyes test, and then the signal detection task. Participants wore over-ear Sennheiser HD206 headphones with the volume set to 20%. The SDT was run using E-Prime 2.0 on a 17″ Lenovo laptop.

### Analysis

All analyses were conducted in R. Group differences for hallucination proneness, signal detection bias (*β*), and social cognition performance were compared using Welch’s *t*-tests. *d*′ (or sensitivity) on the SDT was also analyzed as a control variable. Prior to analysis, log-transforms were applied to LSHS scores and *β* scores on the SDT, while a square root transformation was applied to *d*′ scores; this reduced skew in the data and served to normalize distributions within each IC group. However, for ease of interpretation, raw scores are included in the reporting of descriptive statistics.

## Study 2: Results

Table 4 shows the mean scores for each IC group on the LSHS, signal detection task, and Reading the Mind in the Eyes task. To correct for multiple comparisons across the main outcomes for the questionnaire and two tasks, the alpha level was adjusted to 0.016 (0.05/3). When the groups were compared, significant differences were evident for LSHS, *t* (18.79) = 2.73, *p* = 0.013, *d* = 0.99, indicating higher hallucination proneness in the IC group.

**Table 4:** Hallucination-proneness and task performance by IC group.

	*M* (SD)	
	IC (*n* = 14)	No-IC (*n* = 24)
LSHS	18.21 (5.94)	13.85 (3.18)
SDT		
- Hits (%)	66.66 (11.47)	53.75 (15.46)
- False Alarms (%)	35.42 (19.25)	14.08 (11.21)
- Beta	1.55 (1.82)	2.77 (2.32)
- *d*′	0.86 (0.60)	1.23 (0.41)
RMET	28.21 (3.51)	28.09 (3.18)

IC, Imaginary companion; LSHS, Launay-Slade Hallucination Scale; SDT, Signal Detection Task; RMET, Reading the Mind in the Eyes Test. Hit percentages are calculated from a total of 36 trials; false alarms from a total of 24 trials.

On the signal detection task, both groups were more likely to say speech was absent than present (as indicated by mean scores over 1), but IC participants showed significantly lower *β* scores than controls (i.e., they exhibited more bias toward responding that there was speech present), *t* (26.92) = 3.00, *p* = 0.005, *d* = 0.96. However, group differences were also evident on the control variable, *d*′, indicating lower sensitivity in the IC group, *t* (17.57) = 2.37, *p* = 0.030, *d* = 0.87. No group differences were observed for scores on the Reading the Mind in the Eyes task, *t* (22.23) = 0.12, *p* = 0.909, *d* = 0.04, n.s^1^.

Finally, although a Fisher’s exact test suggested that the distribution of gender across the two groups did not deviate from parity (*p* = 0.741), we compared LSHS, beta, and *d*′ scores by gender to gauge their potential influence on IC group differences. No gender differences were observed, with the closest to significance being beta scores, *t* (38.04) = 1.71, *p* = 0.09. As this was in the direction of males showing more bias toward reporting speech to be present (*M* = 1.83) than females (*M* = 2.74), with a majority of males being in the non-IC group, this seemed unlikely to have affected the difference observed between IC groups in response bias.

## Study 2: Discussion

Study 2 presented us with the opportunity to investigate associations between IC status and hallucination proneness in the context of measures of relevant cognitive processes. We replicated Study 1’s finding of higher hallucination proneness in the group of adults with childhood ICs. Our findings also aligned with previous results showing a relation between hallucination proneness and bias (*β*) on an auditory signal detection task, with participants in the IC group showing a greater bias toward responding that speech was present. We did not replicate the previously observed finding of no differences in sensitivity between groups high and low in hallucination proneness; in our sample, participants in the IC group showed reduced sensitivity. This is in line with a few studies that have reported patients with AVH showing reduced sensitivity as well as bias (e.g., Vercammen et al., 2008). The two IC status groups did not differ on social cognition (theory of mind) performance, suggesting that the group effects on cognitive task performance were specific to the signal detection task.

One limitation of Study 2 was the small sample. However, our methodology did require recruiting people with ICs into a lab-based study, as well as requiring parental verification, which made recruitment more challenging. Our findings form part of a small but growing body of research into the neglected area of cognitive processes in adults with a history of ICs (e.g., Firth et al., 2015). In addition, despite our relatively small sample, our findings are in line with previous work on the cognitive processes implicated in hallucinations, with, for example, very similar false alarm rates in the no-IC group compared to those observed in previous studies (Moseley et al., 2014, 2016).

## General Discussion

The two studies reported here were motivated to explore several hypotheses concerning the relations between ICs and hallucinatory experiences. In Study 1, a large sample of online respondents were asked about their experience of ICs in childhood and adulthood. In line with predictions, experience of ICs was associated with a greater susceptibility to AVH, with the highest scores for AVH proneness observed in individuals who had both had an IC in childhood and continued to have one in adulthood. The inner speech reported by individuals with ICs was more likely to include social-like qualities such as dialogicality, other people, and evaluation/criticism. Study 1 also presents the largest dataset yet gathered on the persistence of ICs into adulthood, with around 7.5% of the sample reporting experience of ICs in adulthood.

Study 2 represents the first attempt to link IC engagement with cognitive processes relevant to hallucination proneness, specifically auditory signal detection and social cognition (theory of mind). Individuals reporting ICs showed a greater bias toward reporting the presence of speech in noise, along with reduced sensitivity. The groups did not differ on theory of mind performance, suggesting that the cognitive tasks effects were specific to auditory signal detection.

Taken together, the two studies reported here are in line with the view that engaging with an IC bears some similarities with psychotic experiences, specifically hallucinations. As noted in section “Introduction,” a small body of research has attempted to explore these relations, including Pearson et al.’s (2001) suggestion that engaging with ICs involves non-veridical percept-like experiences, and Fernyhough et al.’ (2007) proposal that engaging with ICs is a by-product of a developmental process involving the gradual internalization of dialogic social exchanges. The present findings are not sufficient either to confirm or disconfirm these theoretical proposals, but they are at least consistent with them. For the first time, the research presented here has been able to relate these experiences to the quality of inner speech, which has been linked both to childhood engagement with ICs (Davis et al., 2013) and to AVH (see, e.g., Alderson-Day and Fernyhough, 2015).

The studies reported here also speak to the question of whether, and how, childhood ICs persist into adulthood. The research described here was cross-sectional rather than longitudinal, and thus cannot address whether the ICs engaged with in childhood were, for those with persistent ICs, identical to those experienced in adulthood. It does, however, suggest that adults who had childhood ICs show cognitive differences from those without such experiences. In other words, the association observed in childhood between IC status and hallucination proneness appears to persist into adulthood.

That is not to say that ICs that emerge in adulthood are underpinned by the same processes that give rise to ICs in childhood. Establishing continuity in IC experience between childhood and adulthood would require long-term longitudinal data, and one should resist the assumption that adult ICs necessarily represent childhood ICs that have not gone away. There may indeed be such continuity, but ICs may also be constructed anew in adulthood, raising the possibility that such ICs are underpinned by separate cognitive mechanisms to those in operation in childhood. This is particularly pertinent for individuals who *only* develop ICs in adulthood: for both hallucination proneness and inner speech, this group were most similar to those who had never experienced an IC at all. It is likely that there are multiple cognitive routes toward hallucination-like experiences in the nonclinical population (Waters and Fernyhough, 2019), especially for those who deliberately cultivate such experiences (Luhrmann et al., 2019). Tulpamancers (Mikles and Laycock, 2015; Veissière, 2015) and spiritualists (Powers et al., 2017), for example, describe non-self, agentic experiences that in some ways parallel ICs, but which often rely on long periods of focused practice (such as meditation). It is possible that such practices could “unlock” ICs for adults who did not otherwise have a childhood proneness or tendency to have IC experiences.

The experience of shaping and engaging with ICs has also been linked to the creative imaginative act of molding fictional characters into existence, where literary writers displace agency into externalized imaginary beings (Taylor et al., 2003; Bernini, 2014). The creation of fictional characters and the generation of imaginary friends arguably share a feeling of distributed agency paired with knowledge of the subjective source of these creative acts. Looking into how readers represent fictional minds can also offer insight into the links between ICs and AVH. There is growing evidence that readers experience fictional voices as highly vivid, personified, and agentive (Alderson-Day et al., 2017; Maslej et al., 2017). Sometimes the personified voices and worldviews of fictional characters even cross into the reader’s experience of the everyday, in what some authors have termed “experiential crossing” (Alderson-Day et al., 2017). This type of crossing between imagination and reality resembles hallucinatory dynamics in terms of the spontaneous emergence of social agents within the mind, thus reinforcing possible links between AVH, the creation and reception of fictional characters, and the experience of ICs.

Data from the cognitive task measures included in Study 2 suggested that there is at least some overlap between the cognitive processes associated with hallucinations and those associated with childhood ICs, supporting the conclusions from self-report measures used in Study 1. Specifically, participants in the IC group were more likely to report the presence of speech in noise than those in the non-IC group in the signal detection task. While Study 1 evidenced elevated levels of inner speech with social qualities (dialogic or evaluative inner speech, or use of inner speech involving other people) in those with ICs, Study 2 suggested that performance on the Reading the Mind in the Eyes Test, an index of social-cognitive processes involved in theory of mind, was not linked to the presence of ICs, suggesting no impairment in mentalizing in individuals with past ICs.

Performance on the signal detection task has previously been linked to an externalizing bias in reality monitoring (i.e., a bias toward misattributing imagined events as real; Brookwell et al., 2013), or over-weighted top-down processes influencing perception (Moseley et al., 2016), suggesting that ICs may be linked to these cognitive processes. However, it is noteworthy that participants in the IC group also showed a lower sensitivity (*d*′) on the signal detection task, indicating that they also were less able to distinguish the speech from the noise. This pattern is divergent from previous studies showing that hallucinating psychosis patients showed a difference in response bias but *not* sensitivity (e.g., Bentall and Slade, 1985; Varese et al., 2012), though some previous studies have reported reductions in both measures (e.g., Vercammen et al., 2008). While a bias toward speech detection may be consistent with reality-monitoring or top-down accounts of hallucinations, a reduction in sensitivity may also indicate more basic perceptual disturbances. Further research is needed to untangle specific patterns of performance and their association with ICs and proneness to hallucinations. Overall, cognitive data from Study 2 support the continuity across age in IC engagement suggested by the questionnaire data in Study 1 – and indicate more of a link with basic perceptual disturbance than social cognition or theory of mind – but at the same time are slightly different from a patient profile (in highlighting differences in sensitivity).

Although the signal detection task is widely used in the hallucination literature, it is possible that alternative tasks might shed further light on the cognitive processes involved (Brookwell et al., 2013). For example, a limitation of signal detection tasks in understanding AVH is that they do not typically manipulate the amount of auditory verbal imagery used by participants in performing the task. Future research in this area might utilize paradigms which can manipulate engagement in such imagery (Moseley et al., 2016). Other reality-monitoring tasks, particularly those drawn from the episodic memory literature, might reveal different associations with the variables of interest (e.g., Garrison et al., 2017). Future research might also consider the role of autistic traits in the observed relations among ICs, AVH, and inner speech. Such traits are known to affect weighting of sensory information (Karvelis et al., 2018), although their relation to ICs is only beginning to be explored (Davis et al., 2018). Although there are practical difficulties with long-range longitudinal research, investigating the development of these traits and abilities over the life course would be highly desirable.

As summarized above, limitations of the present study include the relatively small effect sizes in Study 1, the embedding of our data collection in a wider study of reading imagery and the use of online self-report (Study 1), and the relatively small size of the sample in Study 2. A further potential limitation of both studies is that recall of childhood experiences might be unreliable (the reason why we sought parental corroboration in Study 2). In addition, it is possible that the presence of AVH is associated with autobiographical memory biases that might increase the likelihood of childhood ICs being recalled.

Notwithstanding these limitations, the present findings provide some support for the view that ICs develop in childhood as a by-product of typical developmental processes. A challenge for future research is to find out more about those ICs that either persist into, or are generated anew, in adulthood, along with the cognitive and neural mechanisms that make continued engagement with ICs possible.

## Data Availability

The datasets generated for this study are available on request to the corresponding author.

## Ethics Statement

This study was carried out in accordance with the recommendations of University of Durham Ethics Committee with written informed consent from all subjects. All subjects gave written informed consent in accordance with the Declaration of Helsinki. The protocol was approved by the University of Durham Ethics Committee.

## Author Contributions

CF, BA-D, AW, and MB conceived the study. BA-D and AW collected the data. BA-D, AW, and PM analyzed the data. All authors wrote the manuscript.

### Conflict of Interest Statement

The authors declare that the research was conducted in the absence of any commercial or financial relationships that could be construed as a potential conflict of interest.

## Footnotes

^1^These analyses were also checked for (1) LSHS auditory items only (in line with Study 1) and (2) group differences following the omission of the two participants who did not recall ICs despite their parents indicting otherwise. As for LSHS total scores, IC participants scored significantly higher for auditory LSHS, *t* (23.72) = 3.78, *p* < 0.001. With the omission of the two participants, group differences were still evident for LSHS, *t* (15.06) = 2.14, *p* = 0.0488, and signal detection bias, *t* (20.09) = 2.58, *p* = 0.018, but no longer for sensitivity, *t* (14.12) = 1.87, *p* = 0.083, n.s.
